# Intersections between syndemic conditions and stages along the continuum of overdose risk among women who inject drugs in Mexicali, Mexico

**DOI:** 10.1186/s12954-023-00815-9

**Published:** 2023-06-24

**Authors:** Pablo Gonzalez-Nieto, Anabel Salimian, Jaime Arredondo, Lourdes Angulo, Alejandra García de Loera, Said Slim, Steve Shoptaw, Mary C. Cambou, Eileen V. Pitpitan, David Goodman-Meza

**Affiliations:** 1 Integración Social Verter, A.C., Calle José Azueta 230, Primera, 21100 Mexicali, BC Mexico; 2 Canadian Institute for Substance Use Research (CISUR), 2300, McKenzie Ave, Victoria, BC V8N 5M8 Canada; 3 Department of Medicine, David Geffen School of Medicine at UCLA, 10833, Le Conte Ave, CHS 52-215, Los Angeles, CA 90095 USA; 4 Universidad Autónoma de Aguascalientes (UAA), Avenida Universidad #940, 20100 Aguascalientes, AGS Mexico; 5 Department of Family Medicine, David Geffen School of Medicine at UCLA, 10833 Le Conte Ave, Los Angeles, CA 90095 USA; 6 School of Social Work, San Diego State University, Hepner Hall Room 119, 5500 Campanile Drive, San Diego, CA 92182-4119 USA

**Keywords:** Women, People who inject drugs, Substance use, Mexico, Stigma, Violence, Overdose, Latin America, Safe consumption site

## Abstract

**Background:**

Research on women who inject drugs is scarce in low- and middle-income countries. Women experience unique harms such as sexism and sexual violence which translate into negative health outcomes. The present work aims to provide insight into the experiences of women who inject drugs at the US–Mexico border to identify social and health-related risk factors for overdose to guide harm reduction interventions across the Global South.

**Methods:**

We recruited 25 women ≥ 18 years of age accessing harm reduction and sexual health services at a non-governmental harm reduction organization, “Verter”, in Mexicali, Mexico. We employed purposeful sampling to recruit women who inject drugs who met eligibility criteria. We collected quantitative survey data and in-depth interview data. Analyses of both data sources involved the examination of descriptive statistics and thematic analysis, respectively, and were guided by the syndemic and continuum of overdose risk frameworks.

**Results:**

Survey data demonstrated reports of initiating injection drug use at a young age, experiencing homelessness, engaging in sex work, being rejected by family members, experiencing physical violence, injecting in public spaces, and experiencing repeated overdose events. Interview data provided evidence of stigma and discrimination toward women, a lack of safe spaces and support systems, risk of overdose-related harms, sexual violence, and the overall need for harm reduction services.

**Conclusion:**

Women who inject drugs in Mexicali describe experiences of violence, overdose, and public injecting. Women are particularly vulnerable in the Mexicali context, as this area faces a noticeable lack of health and social services. Evidenced-based harm reduction strategies such as safe consumption sites and overdose prevention strategies (e.g., naloxone distribution and training) may benefit this population. Evidence from local organizations could help close the gap in service provision in low-resource settings like Mexico, where government action is almost nonexistent.

## Background

Women who inject drugs are at high risk for negative health outcomes such as human immunodeficiency virus (HIV), hepatitis C virus (HCV), sexually transmitted infections (STIs), and overdose [1–3]. The combination of stigma, gender-based violence, and relationship power dynamics with intimate partners increases harm for women [4–6]. These factors may translate into elevated risk-taking, such as engaging in transactional sex work. Stigma and gender-based violence also create barriers to accessing healthcare, including harm reduction services [7, 8]. Experiencing multiple types of violence, specifically sexual violence, has been shown to amplify the risk of overdose in women [9].

Research and interventions specifically targeting women are scarce and are mainly situated in high-income countries [5]. The current study was set in Mexicali, the capital of the state of Baja California, Mexico, adjacent to the US–Mexico border. During the study period, there was one harm reduction service provider in the city: “*Integración Social Verter A.C.”* (herein “Verter”). This non-governmental organization (NGO) operates various harm reduction services, including a needle-syringe program (NSP), a naloxone distribution service, and a women-only unsanctioned safe consumption site (SCS)—the first of its kind in the Global South [10]. The high prevalence of injection drug use in the region is well-documented and is further complicated by issues surrounding deportation, immigration, and violence at the US–Mexico border [11, 12]. The introduction of fentanyl to Mexico’s northern border, documented first in 2018, has produced an unstable opioid supply which further increases the risk of overdose for people who use drugs [13, 14]. The flux in Mexicali’s opioid drug market, related to the introduction of fentanyl, was documented in women who accessed Verter’s harm reduction services [15].

Overdoses in Mexico are a current but neglected public health emergency concentrated along the US–Mexico border [16]. A few studies have documented overdose risk factors and correlates in Tijuana [17–19] and community overdose response in Mexicali [20]; however, the topic remains understudied. In a baseline analysis of *El Cuete IV* data in Tijuana, the proportion of women that reported a non-fatal overdose in the 6 months prior to enrollment was higher than men (12.4% vs 7.8%, *p* = 0.05) [19]. However, during two years of follow-up, there was no difference in reported non-fatal overdoses between genders (women, 44.3%; men, 55.7%; *p* = 0.2) [18]. No studies have explored the relationship between gender and overdose in this region.

This research was informed by the theory of syndemics and the continuum of overdose risk (COR) framework [21–23]. Syndemic theory argues that marginalized groups, like women who inject drugs, due to intersections of biological, social, and structural conditions, are at disproportionate risk of experiencing negative psychosocial and health outcomes such as gender-based violence, adverse mental health, substance use, HIV, and other infectious and chronic diseases. We employed the theory to inform our initial inquiry regarding the types of factors, or syndemic conditions, that women may experience and that may impede their access to harm reduction services. Given our focus on a group of women who inject drugs at risk of overdose and that described experiencing and witnessing overdoses, we used the COR framework to explore factors that may modify the risk of fatal overdose for this subset in Mexicali.

The COR framework, introduced by Park et al., reframes the social determinants of health to highlight key stages of risk along the trajectory of initial drug use toward fatal overdose, as well as proposing six strategies to de-escalate risk of fatal overdose. The five stages of risk in the COR are (1) drug use initiation, (2) active drug use, (3) addiction, (4) non-fatal drug overdose, and (5) fatal drug overdose. The six de-escalation strategies are (1) meaningful partnerships with people who use drugs (PWUD), (2) prevention, (3) harm reduction, (4) treatment, (5) recovery, and (6) reversal of the criminalization of PWUD [23]. We framed our analysis using these stages. Informed by both frameworks, our objectives were to explore barriers and facilitators of harm reduction access and overdose risk among an under-researched group of women in the Global South. Specifically, we aimed to identify the syndemic conditions they experience, and that intersect with and provide context around the different stages along the COR.

## Methods

This was a secondary analysis of a pilot study that aimed to understand the specific needs and vulnerabilities of women who inject drugs in Mexicali in the context of Verter’s harm reduction services [15, 20]. A previous article using data from this study describes results related to behavioral changes after samples used within the SCS were tested for fentanyl [15]. From December 2020 to February 2021, staff recruited women using a purposeful sampling strategy based on recent injection drug use. Participants were recruited while accessing Verter’s harm reduction or sexual health services or during staff mobile outreach activities.

The study included a quantitative and a qualitative component; participants could complete either component or both. The design of the instruments was guided by the syndemic theory and aimed to capture the intersection of syndemic conditions. To be eligible for the qualitative component of the study, participants had to: (1) identify as a woman, (2) ≥ 18 years of age, (3) have injected drugs in the past 30 days, and (4) be able to provide informed consent. To be eligible for the quantitative survey, women also had to (5) plan to use safe consumption space to inject drugs, and (6) be willing to provide a wrapper or filter of intended drug to be used within the safe consumption space. Participants were excluded if (1) they were acutely intoxicated, or (2) were experiencing pronounced withdrawal symptoms that would prevent survey completion, or (3) if using the SCS put individuals at risk. For this analysis, we included only those participants who completed both the qualitative and quantitative components of the study. The UCLA Institutional Review Board (IRB) and the Prevencasa, A.C. IRB approved the study protocol.

For the qualitative component, participants were interviewed by a female co-author (AG) in a private setting. Since data were collected during the COVID-19 pandemic, interviews were conducted over Zoom and only audio was recorded to help promote participant comfort. Most sessions lasted nearly one hour. The interview guide was informed by our theoretical frameworks and included questions and probes around women’s drug use experiences and history, stigma, sexual violence, and experiences using harm reduction services and overdose [20]. Interviews were conducted either in Spanish or English based on participant choice.

For the quantitative component, participants completed a computer-assisted survey instrument via REDCap. Survey topics included socio-demographics, injection use patterns, and questions related to women’s access to harm reduction services. Stigma was evaluated using questions from the Substance Use Stigma Mechanism Scale. The survey was either in Spanish or English based on participant choice. Women who completed the quantitative component used the SCS and fentanyl test strips to test samples intended for consumption within the SCS. Participants completed the interview or survey within 2 weeks of each other if completing both.

All participants were given a safe injection kit (which included sterile needles and syringes, a tourniquet, a clean “cooker,” a cotton filter, alcohol swabs, and internal and external condoms), an overdose reversal kit (which contained two ampules of naloxone, two needle-syringes and a rescue mask), and compensation of $10 US dollars (or its equivalent in Mexican pesos) for each component completed.

Verbatim transcription of interviews and subsequent translation were completed by trained staff proficient in English and Spanish (AG and PG). A co-author (JA) checked all transcripts and translations for correctness. Transcripts were encrypted and uploaded to a secured server. An initial coding guide was developed informed by the interview guide and theoretical frameworks, and Atlas.ti software was used to code translated transcripts.

Qualitative data were examined employing a thematic analysis approach [24, 25]. After coding the transcripts guided by the syndemic theory, as per the design of the instruments, themes were generated and arranged to highlight the stages described in the COR framework. Syndemic conditions that may influence or produce certain stages of the COR and their overlaps were highlighted. Quotes that exemplified each COR stage and de-escalation strategies were selected and presented. We did not follow a mixed methods, triangulated study design and rather chose to focus on each data source separately—on themes that relate to the study objective and utilizing descriptive statistics as complementary data to the in-depth interviews. Quantitative data were analyzed using R version 3.6.2 (Vienna, Austria) and are presented intertwined within each salient theme.

## Results

### Descriptive

In total, 35 women completed at least one component of the study. For this study, the 25 participants who completed both the computer-assisted survey and interview were included in the analysis. Descriptive statistics are shown in Table 1. The median age was 35 years (IQR 28–42 years). One woman self-identified as transgender. Women born in Mexico accounted for 84% of the participants, as well as three women born in the USA, and one woman born in Honduras. The marital status of 36% of participants was common-law union, and 64% were single or had never been married. Nearly half of the participants (40%) had graduated from middle school, and only 16% had graduated from high school. More than half of the participants (56%) considered themselves homeless. Regarding sources of income in the past year, 12% of women reported sex work, 24% had help from family and friends, 4% were employed in another kind of illegal activity, 36% completed informal work, 16% reported legal paid work, and 24% responded income from other sources. More than one option could be selected.

**Table 1:** Descriptive statistics of 25 women who use Verter’s harm reduction services in Mexicali, Mexico (*n* = 25)

Variable	*N* (%)
Age (median (IQR))	35 (28–42)
Transgender woman	1 (4)
Country of birth	
USA	3 (12)
Mexico	21 (84)
Honduras	1 (4)
Marital status	
Single/never married	16 (64)
Common law marriage	9 (36)
Highest academic degree achieved	
High school	4 (16)
Middle school	10 (40)
Elementary school	5 (20)
None	6 (24)
Experiencing homelessness	14 (56)
Main income source in the past year	
Legal paid work	4 (16)
Informal work	9 (36)
Another illegal kind of work	1 (4)
Sex work	3 (12)
Help from family and friends	6 (24)
Other	6 (24)
None	0
Reported drug use in the last three months (%)	
Fentanyl	4 (16)
Heroin	7 (28)
Methamphetamine	19 (76)
Benzodiazepines	14 (56)

### Injection drug use initiation and mental health

The women entered the first stage of the COR, drug use initiation, at an early age. The median first age of injection was 17 years (IQR 15–24) with a range of ages between 12 and 36. More than half (52%) of the participants reported being under 18 at the time of their first injection. The most common drug that was injected during initial use was heroin (60%), followed by methamphetamine (20%), and both drugs combined (8%). For the first-time injecting, eight women had no help (32%), seven were helped by a friend, four by a sexual partner, three by their spouse, and three by a family member.

The women described causes for injecting such as their isolation, family-related issues, and the desire to escape situations in their life. This suggests that injection initiation may have been motivated by coping mechanisms to deal with life difficulties. Further, participants also described wanting to inject and requesting close-by people who inject drugs (PWID) to assist them, highlighting that young women who have close contact with other youth who inject may be more likely to begin injection practices. These quotes allow us to observe the motivations behind injection initiation for women and the role of substances as a coping strategy against adverse circumstances. These factors prompt injection initiation, contributing to the syndemic conditions that increase the likelihood of negative health outcomes and increased risk along the COR.

“… I started when I was 20 and my mother-in-law had taken my daughters from me and I saw that my partner was injecting with his friends, and I said: ‘I want, I want to try, I want to know what's up with this, because I saw how they fell asleep, at ease, and I wanted to forget about it, just like them... and yes, I did like it, because yes, yes it anesthetizes you.” (Participant 5, Age 29)

“I began 6 years ago, I grabbed it [heroin] because I wanted to forget about things, to forget some things, and I asked a girl who was injecting herself if she could help me inject” (Participant 17, Age 27)

“I began to use heroin at the age of 24 and the motive that caused me to start using was well... because of a lack of support from the family, at that time I was working as a sex worker, so because of issues with my family. My mother also worked as a sex worker and well the lack of her being with me, I am the only woman, and I am the youngest and the only one who has been on the streets more, I am the only one who likes to use heroin. That was the reason why I started using heroin, I took refuge in a substance.” (Participant 6, Age 39)

### Active drug use and social stigma

Using drugs led to rejection and discrimination from the wider society, family relations, and healthcare personnel. Substance use was described as a cause for rejection from other social circles, resulting in the seclusion of groups of PWID and the isolation of women, with 84% of participants reporting being treated differently due to their alcohol and/or drug use history and 56% reporting being rejected from employment due to drug use. Participants described internalized stigma, with 64% of the participants reporting “agree” when asked if they “feel like a bad person” and “feel they are not as good as others,” as well as 72% that reported “feeling ashamed” due to their alcohol and/or drug history.

The effect of stigma on the immediate reality of these participants is exemplified by descriptions of common societal beliefs against PWUD; associations of illicit substance consumption with criminal behavior and illness are portrayed. Notably, the quotes exemplify a higher severity in the consequences of stigma for being a woman, highlighting that they are held to a higher moral scrutiny because of their gender and age, while men experience less societal consequences from using substances.

“… before, when I was walking down the street, they looked at me with respect, they greeted me, they even laughed with me. And now that I'm going down the street, they stand to one side, they start pointing at me or there are times that they even call the police on us because to them we are disgusting, they do not want us, they do not want people like us anywhere because we are a bad influence for all young people and all people, because we don’t have the age, we don’t even have the gender to be able to use that substance. And yes, I have suffered a lot of discrimination, all of that.” (Participant 13, Age 24)

“I think people treat me differently because of my physical appearance and the fact that they know I use, people stay away a bit more and take you less seriously, everyone screws you over, and we all have the same reputation of being untrustworthy or being sick and things like that. That’s why people when they see you, the first thing they think about is a ‘tecato’ [local label for PWID] and it’s the worst, when we all deserve to be treated well, but…” (Participant 9, Age 27)

“… if they see you injecting on the sidewalk, which you see frequently around here, one looks worse than a man, truly” (Participant 8, Age 57)

Stigma and discrimination in healthcare settings, as well as perceptions of medical ill-treatment and neglect, hindered the women’s access to care which increases risk by contributing to syndemic conditions. Participants reported maltreatment from medical personnel, with 20% reporting having to stop going to these services. Further, 88% of these women felt as though it is likely that healthcare workers will not listen to their concerns due to their alcohol and/or drug use history and believe healthcare workers will give them poor care. Qualitative data complement these findings by describing life-threatening perceptions of available health services and subpar quality of treatment, often involving deficient pain control measures and unnecessary procedures.

“… so here in Mexicali, for example, in the general hospital they have a green light to kill all the people that are ‘tecatos’, because in less than two months they killed 6 of my friends. One of them was shouting for his mom to get him out because they were going to kill him. And that’s what happened, they killed him, he came out of there feet first” (Participant 1, Age 43)

“Ah yes, in the hospital they discriminate against you a lot and they talk to you very ugly. Yeah, in the government health centers they are really mean, that's why when I get sick I prefer to draw the line and have them treat me over there (in the U.S.), because here they are... here many people say that they kill you in the hospital and I think so, because many friends have gone being fine, only to be treated for a “ cuerazo” (an abscess) they have, just to have it healed and cleaned and they end up coming out feet first, they say they had an attack, that this or that happened… and I think oh, this is true, they kill them. I didn’t believe that before but I do now, a lot of strange things have happened.” (Participant 8, Age 57)

“In the Red Cross and in the hospital, you get discriminated against if you go with a ‘cuerazo’ (abscess), if you complain in the slightest, they kick you out and leave you there, they also cut you up badly even though it’s not needed” (Participant 6, Age 39)

Accounts of rejection and discrimination by family relations were prevalent. Most participants (75%) reported being rejected by their families because of using substances: 64% of women reported often being looked down on and treated differently by family members due to their drug use. When anticipating how likely it would be that family members would think they could not be trusted, 64% responded it was very likely. Testimonials describe rejection and negative judgement by family networks and appear to reveal a causal relationship with participants searching for stigma-free social circles and spaces, often around other PWID. Harmful perceptions of substance use are seen to be recreated within the family sphere, leading to a decreased sense of self-worth within those relationships and the continued marginalization of the women. These conditions increase the risk of overdose along the COR as they appear to contribute to active drug use.

“P: Because my family ... they call me ‘tecata’ and it's like the worst, it’s the worst for them […] it's like you are disgusting or they think that I'm sick because of that or things like that […]

Interviewer: So, you feel like your family has pushed you aside, but is it precisely because of that [using substances]?

P: Precisely because of that.” (Participant 9, Age 27)

“I started to be more on the streets, to ask for money, clean cars, everything that a person who uses substances does, live on the sidewalk, in empty lots or abandoned houses. Family doesn’t accept you anymore, they don’t trust you, bit by bit the trust goes away and you lose the family’s support.” (Participant 14, Age 38)

### Active drug use and violence

Physical assault, sexual violence, and transactional sex were prevalent experiences among participants, often in relation to substance use. Physical abuse was common, with 32% of women reporting being beaten by their intimate partners. Women described experiencing a high degree of sexual violence, with 29% percent of participants reporting being raped in the last three months. When asked if they were ever provided with a good or service in exchange for sex, 57% of participants answered “yes.” The most common exchanges were money (100%), drugs (58%), clothes (42%), and gifts (50%). With restricted access to these resources, women are faced with substantial personal risks. Accounts of sexual assault were also common, including at a young age. The quotes depict rape while being unconscious after using substances, forced exchanges of sex for drugs by intimate partners, and more violence linked to the use of substances, with some participants blaming violent experiences on using drugs.

“… you get to live many experiences, I’ve used, I’ve gone unconscious, and they have done many things to me [while unconscious], I have stood up when they were finished doing it, and without using a condom or stuff like that, and so it’s just having to come [to Verter] and get checked and wait to see if you’re okay and if the man is okay, and that’s it” (Participant 9, Age 27)

“For example, I lived with a man like 5 years ago, and with that man, we didn’t have money for the drug, right? And I was pregnant, I had 4 months. So, because I didn’t want to go be with one of his friends to get money for the drug, he beat me, and beat me, and beat me, until he almost killed me.” (Participant 3, Age 27)

“Well yes, I have been physically hurt before, and also mentally, and well everything is because of that, the addiction, if I didn’t have that addiction nothing like this would happen to me, because the circle I hang out with are all thieves, rapists, murderers, the worst of it all” (Participant 16, Age 68)

### Addiction

Addiction is the third stage along the COR. All 25 women agreed with having a drug problem and 92% self-identified as an “addict.” The majority (88%) acknowledged that if they do not make changes to their drug use soon, their problems will only get worse. Additionally, 72% of women reported wanting to make changes to their drug use, with 76% of women reported starting to make changes in their drug use, and 68% of women say they are actively doing things to cut down or stop their use of drugs. However, the difficulty of cutting down on consumption is linked to a lack of resources and quality treatment, and other syndemic conditions that contribute to the prolongation of active use.

“… with drugs, I don’t know if I can stop, really, because since I was very young, since I was able to think, I use drugs. And sometimes it gets really hard, I already had like two or three months without using and I go back, and the first thing I think about is to go and use drugs.” (Participant 4, Age 44)

“No, no I don’t really feel like doing a lot, I want to leave it, but for the ‘malilla’ (withdrawals) I can’t leave it because I start to feel bad. But in my heart, I want to stop […] I’m getting it down a lot. I want to go down to one, little by little stop completely.” (Participant 12, Age 54)

### Fatal and non-fatal overdoses

Many syndemic conditions seem to contribute to the last stages of the COR, non-fatal and fatal overdoses. The changing environment in the local drug supply related to the introduction of fentanyl made experiences related to opioid overdose common for the women. Participants highlighted how, because of being a woman, certain overdose-related risks were exacerbated, including being sexually assaulted. Almost all (76%) reported having suffered an overdose in their lifetime. When asked if they had used any drugs known or now believed to contain fentanyl in the last 3 months, 76% responded yes. Further, when asking about participants’ ability to access fentanyl, 75% said they could get it the same day. The majority of women (85%) reported that the last time they obtained fentanyl it was sold as black tar heroin.

Out of 25 participants that used the SCS and had their samples tested for fentanyl, 15 of these women’s samples tested positive for fentanyl. Multiple participants retold traumatic experiences of overdose and naloxone administration, either involving themselves or their peers. Testimonies of peers as first responders were frequent within qualitative data, with mentions of community members suffering fatal overdoses also being common.

“They leave me there, they give me two, three slaps, and because they see that I don’t wake up they leave me there, later in the evening I wake up, alone, where I did the shot, without my stuff, and maybe they did something to me, they might be my addiction partners and whatever you want, but here it’s everyone by themselves, you get me? And then imagine being a woman.” (Participant 14, Age 38)

“It makes me scared, I have never overdosed, I don’t know what it feels like to overdose, but I have been there when people go down and I have brought them back, I’ve come to get the injections (naloxone) here (Verter) and they come with me and help me, they don’t let anyone die” (Participant 8, Age 57)

“Yeah, I know fentanyl. I have a lot of friends that have passed away because of it, that have ‘doblado’ (overdosed). Yeah, it’s like it’s stronger or I don’t know but, whatever it has, it’s the same amount that they were using before and they die, so it’s really hard.” (Participant 9, 27)

### Harm reduction services

Harm reduction is the first de-escalation strategy proposed by the COR that was present in the themes; these services have historically been a link between PWUD and health services. Participants highlighted the lack of safe spaces and services, with Verter being one of the few available to them. Many women described the ways in which they benefit from Verter’s services. For instance, despite syringe sharing being uncommon, with only 6 women partaking in this practice in the last three months, 96% of women acquired their clean injection equipment from Verter.

When asked about naloxone access, 67% of women reported having a take-home naloxone rescue kit. All but one of the women who had naloxone reported getting it from Verter. Before coming into the interview session, 28% of participants had never heard of naloxone rescue kits. Twelve participants (67%) had administered the drug to another person in the past three months, and 28% reported they had been administered naloxone due to an overdose in the same period.

Related to drug checking services, 20 women reported that they had tested their drugs for fentanyl in the last three months. All of the women that did not use this service said they did not know of the availability. However, a majority of these women (65%) reported only using it less than once a month, with 15% using it one to three times per month and 15% using the services two to three times per week. Women reported using fentanyl test strips at the drug checking service, and in doing so, 50% of these women reported a change in their decision to use/how to use based on the result. Due to the presence of fentanyl, nine women decided not to use alone, and one woman chose to use by other means, not by injection.

The quotes exemplify how Verter’s services limit instances of overdose within their local environment and minimize the risk of syndemic conditions like sexual violence, law enforcement, and discrimination. These interventions capture several de-escalation strategies along the COR, contributing to lowering overall risk. The severe lack of services catered to this population in the city is also made explicit through these narratives.

“I have come to ask for help like this sometimes that I cannot find another place to run to, other than here [Verter], which is where I feel that they help me, they support me, because since I was 13 years old, I have had to get treated with a psychologist.” (Participant 13, Age 24)

“… they can test you for everything, AIDS, hepatitis, everything, so you can know if you’re clean or not, and you can use there […] being certain the cops won’t take you away, you get me?” (Participant 3, Age 27)

“I come here for syringes, for the exchange, to be with the other women, chat with them, share my experience, or like to see if you don’t have any diseases, how to put a condom on and all that […] or how to use the little flasks (naloxone)” (Participant 26, Age 41)

“Yeah, I prefer to come here than getting picked up by the cops or that they come into the ‘yongo’ (empty lot) while we’re using and they get us. So, I come to the SCS and I feel comfortable, I’m relaxed, and they even give me a coffee” (Participant 16, Age 68)

### Treatment

Experiences with substance use treatment were shared among the majority of participants; however, the participants’ accounts reflect the dire need for quality and low-barrier treatment services. Efficient services would help reduce the risk of fatal overdose for these women. Twenty-two women reported receiving professional help for the use of alcohol or drugs. Out of those women, 77% reported being enrolled in a methadone program in their lifetime; however, only three women reported being enrolled at the time of the study interview. Reasons for stopping methadone treatment included price, having spouses/partners that were not undergoing treatment, and reports of using other drugs in combination. The emergence of fentanyl shifted perspectives in 36% of women as they reported wanting to access addiction treatment in the near future. One woman reported already accessing addiction treatment due to concerns about the emergence of fentanyl. Women reported reasons for not accessing any kind of help or treatment to be due to fear of withdrawal, financial cost, dislike of the treatment regimen, and a location out of proximity. Involuntary treatment was common among participants and was also listed as a reason for treatment ineffectiveness.

“Interviewer: So, you told me that your intention is to stop using heroin completely. Have you ever been in treatment to stop using it?

P: Yes, I was in a rehabilitation center a while ago, but I left because it wasn't my intention, it wasn’t something I wanted, I was forced and what I actually wanted was to continue to use. I went out [of the treatment center] and came out to the same thing, but this time I'm kind of tired, I think now would be a good opportunity to leave it [heroin], because the other day I didn’t have any, and you get the cravings, and it would be good to leave it.” (Participant 9, Age 27)

“It (the treatment center) was good, they gave you time to recover and then you get psychotherapy, you talk and express yourself, and I think it’s good, it’s like therapy, I think that’s good, no?” (Participant 4, Age 44)

“P: … one day that I didn't have money for methadone, I felt worse than when I got ‘malilla’ (withdrawals) from heroin, you know what I mean?

Interviewer: Yes, it was worse, right?

P: Aha, and that’s why I said no, I’m going to cut it off here, because they told me that was worse than heroin. And I better cut it off... And well, money, I spent 200 pesos a day anyway.

Interviewer: Ah, so money was also one of the reasons why you left that type of treatment, right?

P: Yes” (Participant 3, Age 27)

### Criminalization

The last de-escalation strategy proposed in the COR is the reversal of the criminalization of PWUD; the women’s experiences provide vivid examples of how criminalization contributes to overdose risk and other harms. Encounters with police were commonly cited: 88% of participants reported being stopped by law enforcement during their lifetime. Out of the twenty-two women that reported yes, within the last three months, 55% reported that their clean syringes were confiscated after being stopped by law enforcement, 50% reported law enforcement demanding money as a form of a bribe, 45% reported that their money or other valuables were confiscated, 5% reported law enforcement confiscating and never returning ID documents, 32% reported standing before a judge, and one woman reported being sentenced to jail time and compulsory drug treatment. Forty-five percent of women reported being beaten (hit, punched, or kicked) by a law enforcement officer(s) within their lifetime. Nearly half of the participants (44%) reported being told they are subject to arrest or detention due to their possession of drugs, despite being within the legal limit.

“P: There were times when I was in rehabilitation for two to three months.

Interviewer: And when you were there, was it voluntarily or did a family member take you?

P: It was the police.

Interviewer: And for example, what was that experience like or why did the police take you to treatment that time?

P: They were cleaning the streets of all drug addicts, they were picking them up and taking them to a center and well, it happened to me. (Participant 5, Age 29)

“Yeah, right here, the police car comes and snatches you, takes your money away and also gets you on the way back, that’s what they get out of you because of the withdrawals you would get inside (jail)” (Participant 1, Age 38)

“Interviewer: Have you been hit by the police?

P: Well, they haven’t hit me, but they do treat me as if to humiliate me for using drugs in a “yongo” (abandoned lot), where we go and use or things like that, for the syringes, they come and they treat you like the worst and it's wrong. I think we all deserve respect.” (Participant 9, Age 27)

We did not identify significant themes or quantitative data for three de-escalation strategies described in the COR framework: partnerships with PWUD, prevention, and recovery.

## Discussion

The data presented prove that this group of women is at great risk of overdose, as it provides evidence for all of the stages along the COR in this population. Our findings highlight the multiple syndemic conditions such as physical violence, sexual maltreatment, stigma related to their substance use, and a lack of services, that these women experience. These conditions contribute to the advancement of individuals toward more severe stages along the COR, increasing the risk of fatal overdose.

As previously described in the literature on women who inject drugs, gender-associated stigma is very common in this population [6]. In our study, participants recognized that being older and male could make the use of substances more permissible. Stigma is also known to increase the marginalization of women who use drugs, hindering their access to services [7] and increasing their risk for serious health complications, such as HIV infection [26]. Offering a safe space free of stigma is a first step toward addressing some of the risks that women experience in the city. However, wider acceptance of drug use on a societal level and views of substance use disorders as public health issues rather than a criminal problem are needed.

Treatment services are limited for women in the Global South, including Mexico. The cost of daily treatment at the local methadone clinics was identified as a predominant theme. Mexico lacks a free option for medications for opioid use disorder, and all private clinics in the country have currently closed [27]. In Tijuana, methadone and related services (e.g., psychosocial support) were estimated to cost between $95 and $179 US dollars per person [28]. Past the cost, stigma is well documented as a barrier for people who wish to receive treatment at methadone clinics, and this is especially prominent for women [6]. Buprenorphine-based treatment in primary care clinics could mitigate some of this treatment-related stigma [29]; however, without systematic government support to make buprenorphine free of charge, an intervention like this is unlikely to be effective for women.

Criminalization was highlighted as a major issue for women. State forces were described to be involved in illegal activities such as theft, extortion, and physical violence. Police violence against people who inject drugs along the border has been well documented along the US–Mexico border [30]. In a Tijuana-based study, need for treatment was correlated with higher arrest and assault rates [31]. An older study depicted how police persecution drove risk practices related to injection in Tijuana and Ciudad Juarez [32]. More recently, a study looked at female sex workers who inject drugs in both cities and suggested that police likely target this vulnerable subgroup [33]. These findings are important, as the majority of the women in this article reported exchanging goods for sex and could indicate increased risk in the city. The human rights commission of the state where Mexicali is located also documented illegal arrests of PWUD in the city and issued a recommendation to the municipal police [34]. The literature that focuses on experiences of women who inject drugs and police interactions along the border is limited, making the Mexicali accounts a significant piece of evidence that highlight the pressing need for policy interventions in the region.

The data also point to the ways in which harm reduction services, including the SCS at Verter, provide a safe harbor for women—not only when they are using, but before and after, when risk of violent experiences is heightened. Analysis of this data reveals key themes along the syndemic and COR frameworks that can inform on valuable interventions for women who inject drugs at the US–Mexico border. The need for support is particularly pressing in the Global South where low-resource conditions seem to be similar to Mexicali, with little to no services for PWUD. In Mexico, the federal government defunded all civil society efforts directed toward harm reduction [35], increasing the risk of PWID by limiting resources and the spaces where they could feel safe and supported.

Increased availability and access to safe consumption sites are needed for women who inject drugs. More than half of the women reported injecting in a public space, and nearly all of them reported overdosing during their lifetime. SCSs can provide a space that is free of gendered violence, stigma, criminalization, and police harassment. These facilities have also been shown to decrease public injection and fatal overdoses [36]. In the context of the US–Mexico border, police forces and people with lived experience have shown acceptance of such interventions [37]. Because of the increased stigma that women who inject drugs experience, having SCSs exclusively for women could prevent risky behaviors by diminishing harm in these women’s current microenvironment [38, 39].

Additional interventions to de-escalate risk along the COR for women are needed in this region. For example, community-based drug checking programs are necessary, so people can know the contents of the substance they will consume and dose appropriately, particularly in a market flooded with illicitly manufactured fentanyl. An appropriate forensic and toxicology reporting system is also needed to gauge the true amount of overdose fatalities in low-resource settings like Mexicali, since there are currently no government data available. Naloxone accessibility and training of medical personnel have also proved to be crucial. A scale-up in availability and access to quality treatment services is needed in the region as current services are inadequate or nonexistent. Exploring the adaptation of programs such as the “do not use alone” campaign, implemented in cities across the USA and Canada, may also prove beneficial. Finally, although seemingly out of reach for contexts like Mexicali, where there is a heavy presence of drug trafficking organizations and associated violence, a safe supply of opioids will be necessary to effectively reduce overdose risk and provide compassionate and equitable avenues of care.

Due to context characteristics, de-escalation strategies around partnerships with PWUD, prevention, recovery, and decriminalization efforts could not be identified. This setting exposes the urgent need for these strategies, not only in Mexico but throughout the Global South. Investment in evidence-based prevention programs, quality recovery paths, and criminal reform, are strategies to reduce overdose in communities at risk.

Our study is subject to several limitations, as it is only reflective of one subgroup of people who use substances in the city, and their experiences and responses might not be reflective of others outside of Mexicali. Further research could be utilized to correlate risk factors to risk behaviors, though beyond the scope of this paper. Studies that focus on subsets of women who inject drugs that may experience increased risk, such as female sex workers and trans women, are also greatly needed.

## Conclusion

Women in this study describe high rates of stigma, marginalization, and lack of support systems. These syndemic conditions further augment risk behaviors such as public injecting and non-sterile syringe sharing, fueling the progress along the stages of the COR. Low-threshold harm reduction services in this context are welcomed by this population, particularly where public funding for these programs is nonexistent. Even though existing harm reduction services are insufficient to cover the needs of women who inject drugs, Verter provides interventions such as naloxone distribution and safe consumption services that seem to contribute to the de-escalation of risk for this group along the COR. Continued research engaging community members and organizations is needed to develop and implement syndemic-responsive and structural interventions which are needed to successfully address drug-related harms in low-resource settings such as Mexicali, including overdose and HIV risk.

## Data Availability

The datasets used and/or analyzed during the current study are available upon request to the corresponding author during the review process.

## Abbreviations

PWID: People who inject drugs
HIV: Human immunodeficiency virus
HCV: Hepatitis C virus
US: United States
PWUD: People who use drugs
SCS: Safe consumption site
